# Raman spectroscopy in biomedicine – non-invasive in vitro analysis of cells and extracellular matrix components in tissues

**DOI:** 10.1002/biot.201200163

**Published:** 2012-11-19

**Authors:** Eva Brauchle, Katja Schenke-Layland

**Affiliations:** 1 Department of Cell and Tissue Engineering, Fraunhofer Institute for Interfacial Engineering and Biotechnology (IGB)Stuttgart, Germany; 2 Institute for Interfacial Engineering (IGVT), University of StuttgartStuttgart, Germany; 3 Department of Thoracic and Cardiovascular Surgery and Inter-University Centre for Medical Technology Stuttgart-Tübingen (IZST) at the University Hospital Tübingen, Eberhard Karls University TübingenTübingen, Germany

**Keywords:** Biosensors, Optics, Photonics, Screening

## Abstract

Raman spectroscopy is an established laser-based technology for the quality assurance of pharmaceutical products. Over the past few years, Raman spectroscopy has become a powerful diagnostic tool in the life sciences. Raman spectra allow assessment of the overall molecular constitution of biological samples, based on specific signals from proteins, nucleic acids, lipids, carbohydrates, and inorganic crystals. Measurements are non-invasive and do not require sample processing, making Raman spectroscopy a reliable and robust method with numerous applications in biomedicine. Moreover, Raman spectroscopy allows the highly sensitive discrimination of bacteria. Rama spectra retain information on continuous metabolic processes and kinetics such as lipid storage and recombinant protein production. Raman spectra are specific for each cell type and provide additional information on cell viability, differentiation status, and tumorigenicity. In tissues, Raman spectroscopy can detect major extracellular matrix components and their secondary structures. Furthermore, the non-invasive characterization of healthy and pathological tissues as well as quality control and process monitoring of in vitro-engineered matrix is possible. This review provides comprehensive insight to the current progress in expanding the applicability of Raman spectroscopy for the characterization of living cells and tissues, and serves as a good reference point for those starting in the field.

## 1 Introduction

**Figure 1:**
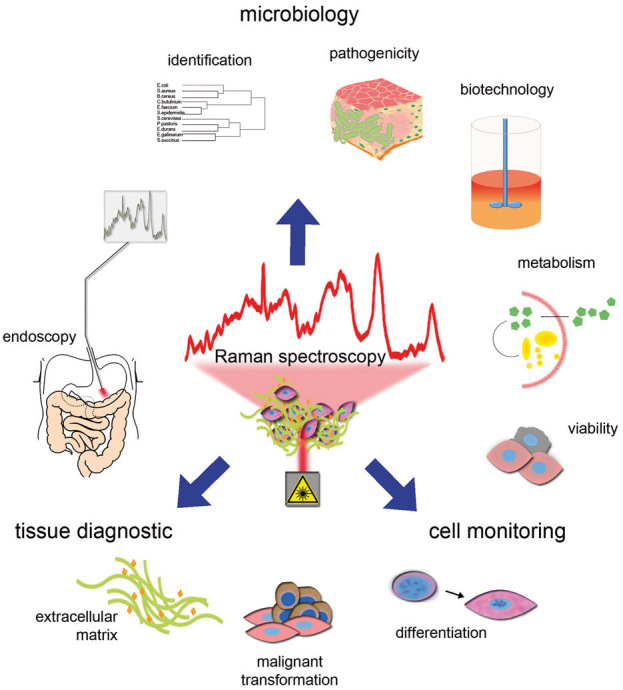
Applications for Raman spectroscopy in biomedicine.

Raman spectroscopy is an emerging laser-based technology that has made its way from physics and chemistry to biomedicine within the past two decades. It can be employed as a non-invasive, non-destructive, and even non-contact monitoring technology in numerous biomedical fields (Fig. 1). Raman spectra of biological specimens reflect their overall molecular constitution including specific signals from proteins, nucleic acids, lipids, and carbohydrates as well as inorganic crystals. Accordingly, living cells and tissues can be identified based on their biochemical signature under physiological conditions [1]. Reliable results can be quickly obtained from minimal sample volumes. In biomedical research, this is a crucial point as biological materials are often rare.

**Figure 2:**
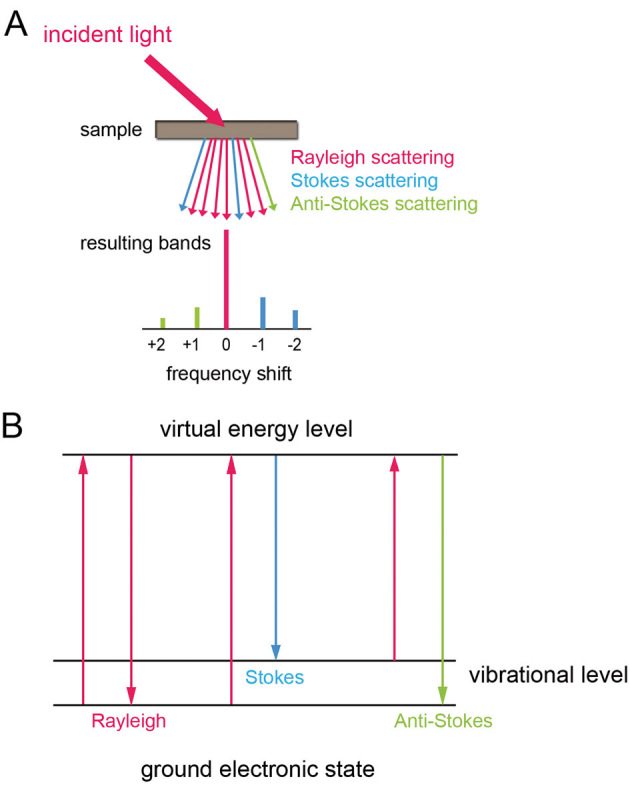
Types of light scattering. (**A**) Raleigh, Stokes, and anti-Stokes scattering and the resulting frequency shift relative to the incident light. (**B**) Molecular energy levels corresponding to the type of light scattering.

The “Raman effect” is named after its discoverer, C.V. Raman, an Indian physician. He first predicted and described the Raman effect as “A new type of secondary radiation” [2]. In his study, he reported for the first time the inelastic scattering of light. Different light-scattering processes arise when photons of light interact with molecules in material. Light scattering is a two-photon process, where one photon is absorbed and another photon is emitted simultaneously. Photons are most often emitted with the same frequency as the incident photon (= Rayleigh scattering), but photons occasionally loose or gain energy due to molecular interactions and are therefore frequency-shifted (Fig. 2A). In Rayleigh scattering, the excited radiation field of molecular electrons matches the frequency of incident photons, thus when emitting the photon, the molecule returns to the previous electronic ground state. In contrast, Stokes scattering and anti-Stokes scattering are inelastic processes where the molecular electrons oscillate in response to the photon excitation [3]. The previous and the resulting electronic ground state are distinct. The emitted photon is frequency-shifted, where the difference to the incident frequency reflects energy that matches specific molecular vibrational frequencies (Fig. 2B). In thermal equilibrium, molecules in the electronic ground state are more frequent, thus photons predominantly experience a Stokes shift [4], which is used in most Raman applications. Overall, only a small part from approximately every 10^6^–10^8^ photons is inelastically scattered [5].

Due to its molecular sensitivity, Raman spectroscopy was first employed to reveal molecular structures of pure chemicals and to date, is used for characterizing and identifying chemical components as well as solid pharmaceuticals [6, 7]. First studies on living cells were performed using a Raman spectroscope coupled to a microscope [8]. Successive studies emphasized that the employment of an appropriate NIR laser beam is crucial to preserve the sample integrity and cell viability [9, 10]. The 785 nm laser diode is currently the most common system used for biomedical applications involving Raman spectroscopy [11]. Multivariate methods are employed for the analysis of Raman spectra [12]. Principle component analysis (PCA) is widely used on spectroscopic data. PCA reduces the spectral variables and reveals peaks that reflect biochemical alterations [13].

This review gives an overview of applications for Raman spectroscopy in the field of biotechnology (Fig. 1). Recent progress in the characterization of microorganisms concerning medical microbiology and biotechnology is included. We also report on the suitability of Raman spectroscopy for the investigation of primary cells, cell lines, stem cells, and pathological cells. Finally, the use of Raman spectroscopy for in vitro detection of collagens and proteoglycans in tissues is discussed.

## 2 Raman spectroscopy and its application in microbiology and biotechnology

### 2.1 Applications in the field of microbiology

Raman spectra of microorganisms can reflect their specific overall biochemical constitution, which is useful for the identification of bacterial species and bacterial strains as well as yeasts. Species classification using Raman spectroscopy follows taxonomic rankings, starting with the differentiation between Gram-positive bacteria, Gram-negative bacteria and yeasts [14]. Raman was previously employed for the identification of several potential pathogens and contaminants isolated from different sources [15–18]. Raman spectra of microorganisms were performed on microcolonies, on solid agar [16], bacterial pellets [19], or single bacterial cells [17, 20]. We were able to discriminate *Escherichia coli*, *Bacillus subtilis*, *Staphylococcus aureus*, and *Candida albicans* based on Raman spectra (*unpublished data*). In these studies, every species revealed a clear distinct biochemical fingerprint. Additionally, we demonstrated that spectra from active *Bacillus subtilis* were completely different to those of spores from *Bacillus subtilis* [21]. Others have shown that bacterial spores are simultaneously distinguishable from other particles in solution [22]. Due to variant concentrations of dicalcium dipicolinate and proteins in the spores of different bacteria, Raman spectra may also help to reveal distinct spores of bacteria species [23, 24]. Raman spectral fingerprinting of microorganisms is robust and highly reproducible even under different conditions impacted by temperature, time, and medium [25]. Accordingly, a Raman-based system for identification of microbial pathogens has already found its way into clinics (River® diagnostics, SpectraCell).

Nevertheless, spectral heterogeneities were observed in presumed pure microbial colonies. Different metabolic states in the middle and at the edge of a colony, or general clonal variability, have been suggested to be reflected by Raman spectra [20, 26]. Clonal variability is of concern in nosocomial infections and their epidemiology. It is well-known that outbreaks of nosocomial infections are due to spreading of single multi-resistant microbial clones [27]. Hence, many Raman studies focused on the characterization of microbial pathogenic factors. Spectral variances in *Listeria monocytogones* were identified due to toxin production [19], as well as ampicillin resistance in *E. coli.* Furthermore, spectral shifts also occurred when ampicillin resistance was activated [28]. Raman spectroscopy has been successfully employed for the investigation of epidemic outbreaks of infections in hospitals, including the analysis of heterogeneity in isolated clones [29–31]. Raman-based characterization has been used in addition to common methods such as serotyping, pulse field gel electrophoreses and genotyping using amplified fragment length polymorphism or 16S RNA sequencing to characterize microorganisms on a subclonal level [18, 19, 30, 32]. Overall, Raman spectroscopy can be considered a straightforward method for the identification of high-risk bacterial strains [33].

### 2.2 Raman spectroscopic analyses in biotechnology

The monitoring of intracellular storage material and metabolic characteristics are critical in the field of biotechnology, where natural or artificial metabolites are purified and serve as industrial products such as chemicals, fuels, or biological active ingredients. Raman-based studies on *Saccharomyces cerevisae* displayed that changes in the growth phases are detectable. Spectral changes were suggested to be associated with the stage of cell cycle and bud formation [34]. Moreover, in biotechnical processes, it is important to obtain information concerning intracellular deposits, as it presents the biotechnical product. Polyhydroxyalkanoate (PHB) is an intracellular storage material that is utilized for the production of biodegradable plastic. Raman spectral investigations focusing on PHB storage in *Cupriavidus necator* demonstrated that intracellular PHB is not only detectable, but the additional monitoring of its accumulation was feasible. Furthermore, it has been shown that the intensity of the PHB-specific peak at 1734 cm^–1^ correlates with the quantification of PHB using classical HPLC analysis [35]. In ethanol fermentation, it must be prevented that ethanol is stored intra-cellular, because it influences the viability of yeasts. Raman spectroscopy is considered a suitable, reagent-free tool to monitor and study process kinetics in ethanol fermentation as it was shown to be sensitive enough to detect intracellular ethanol accumulation in yeasts by the ethanol-specific Raman band near 881 cm^–1^ [36, 37]. Intracellular-produced fatty acids were also detectable using Raman spectroscopy. Although, it was not possible to resolve single fatty acids in bacterial spectra, the spectral fingerprint reflected at least the relation between saturated and unsaturated fatty acids [38]. In different algae species, the Raman spectra reflected their characteristic lipid composition that was successfully correlated to the overall amount of C–C double bonds [39]. Moreover, Raman spectroscopy was employed to screen the complex metabolic characteristic of transgenic cells. In *E. coli* and *Pichia pastoris* yeast cells, which were transfected with an inducible system in order to express somatolactin-β, Raman spectra displayed an increase of specific protein bands when the expression of somatolactin-β was activated. Furthermore, it has been shown that Raman spectroscopy-based quantification of intracellular somatolactin-β in *E. coli* is realizable [40]. Similar results were found in recombinant *E. coli* overexpressing the extracellular domain of an oligodendrocyte glycoprotein [41]. More detailed Raman spectral analyses were performed on nuclei of transgenic cells of loblolly pine. Two cell lines were generated employing viral transfection with two distinct reporter genes. Finally, both cell types were successfully separated based on their spectra [42].

## 3 Label-free cell identification and characterization

### 3.1 Discrimination between primary cells versus cell lines

Similar to the findings in microorganisms, it has been shown that eukaryotic human cell types are represented by a specific Raman fingerprint spectrum. Cellular function and metabolic characteristics contribute to this spectrum, which reflects the uniqueness of every cell type [43–45]. Several studies discussed in this section focused on Raman spectral differences between primary cells and their corresponding cell lines. In our previous studies, we showed that the discrimination between human HaCaT keratinocyte cells and primary keratinocytes is feasible using Raman spectroscopy. HaCaT cells displayed overall higher Raman signals, accounting for protein and DNA, when compared to primary keratinocytes, which may correlate to the higher proliferative activity of HaCaT cells [43]. Herein, it should be noted that the proliferative potential is not necessarily related to a shift in distribution of the cell cycle phases, in which a decrease in signal contribution of lipids can be detected [46, 47]. When the proliferative activity of HaCaT cells is inhibited due to modulated culture conditions, reduced relative protein and nucleic acid intensities were detected [48]. Another comparative study employing Raman spectroscopy, focusing on a A549 human lung carcinoma cell line, primary human alveolar cells, and retroviral-transfected alveolar cells, emphasized that A549 cells are not an appropriate replacement for primary human alveolar cells. In contrast, retroviral-transfected alveolar cells were confirmed to be biochemically much closer to their primary counterpart. Nevertheless, the authors stated that the differences detected between the immortalized alveolar cells and primary cells are directly correlated to the higher proliferative rate, which is especially noticeable in the nucleic acids bonds [49]. Analogous analyses were performed using primary osteoblasts and retroviral-immortalized osteoblasts [50]. Here, no differences between primary cells and the corresponding cell line were evident when performing PCA on the spectral data. In contrast, the osteosarcoma cell line MG63 was significantly distinguishable from primary cells [50].

Raman-based investigations were further performed to test the suitability of the osteoblast-like cell line U20S for its use in tissue engineering. Osteoinduction, which is detectable via an increase of the 960 cm^–1^ hydroxyapatite-peak due to mineralization, was comparable in both U20S cells and primary isolated osteoblasts. Spec-tral characterization of both undifferentiated cell types showed unambiguous differences. Furthermore, Raman spectra showed that the U20S cells underwent spontaneous differentiation due to prolonged cell culture times [51]. Analogously, we monitored in vitro dedifferentiation in primary chondrocytes [52]. Successive passaging of primary chondrocytes resulted in a loss of essential intracellular lipid vesicles that are characteristic for the primary cell phenotype. Loss of lipid vesicles was correlated to the decrease of lipid-specific Raman signals at 1065, 1079, and 1300 cm^–1^. Additionally, we examined Raman spectra of primary chondrocytes versus the SW1353, a chondrosarcoma cell line, showing once more that the cell line exhibited a significantly higher intensity of the Raman spectra [52]. However, since SW1353 cells are malignant in vivo [53], Raman spectroscopy may also be suitable to detect the grade of malignancy. Details of using Raman spectroscopy for detecting pathological cell states are discussed in the next section (section 3.2).

### 3.2 Identification of pathological cell states

Raman spectroscopy was quickly explored as a potential tool for the analysis of pathological cell states. Omberg et al. [54] performed a proof-of-principle study in which Raman spectra were taken embryonic rat fibroblasts that were either immortalized with c-myc or were immortalized and additionally transfected with a ras-oncogen, which led to excessive and aggressive tumor formation in vivo. Raman spectra displayed differences between the two cell lines based on proteins and lipid signals due to malignancy [54]. Similar results were obtained in studies on neuronal and prostate carcinoma cells [55, 56]. In lung cancer cells with different grades of malignancy, Raman spectra were excited using a laser wavelength of 532 nm. Here, a connection between the cytochrome c bands at 749, 1129, 1314, and 1583 cm^–1^ and the increased energy consumption of cancer cells was suggested [57]. Extensive investigations were performed on human epithelial breast cancer cells. It was demonstrated that an increased signal contribution of DNA is connected to malignant transformation. However, in this study, lipid spectra were not included into the spectral peak fitting approach [58]. Other Raman-based studies focused on the detection of metastatic cells. The potential to form metastases was suggested to be reflected by Raman peaks at 1260, 1297, 1400, and 1660 cm^–1^, which were detected in the cytoplasm of the metastatic M4A4 cells [59]. Similar results were found in breast cancer cell lines with a distinct expression of the Her2/neu receptor. Although there was no correlation between the amount of Her2/neu expression and the spectra, the spectra showed a variability in polyunsaturated fatty acids that may be connected to the grade of malignancy [60].

Viral infection is another known player in cancer development; especially in cervix neoplasia. First studies using Raman spectroscopy investigated the cellular influence of high-risk human papilloma virus (HPV) infections. Spectra of the HPV-infected keratinocytes were discriminated from healthy keratinocytes, mainly based on protein-assigned peaks [61]. Subsequent studies examined four different cervix cancer cell lines, each with a different copy number of the HPV genome. Non-infected cell lines and cell lines with low copy numbers were more similar than spectra of highly pathogenic cell lines [62].

Raman spectroscopy was further explored for the diagnosis of cells that were infected with Kaposi's sarcoma-associated herpes virus. Infected hematopoietic cells and their non-infected counterparts were investigated. It was first shown that the Raman spectra of non-infected and infected cells occurred in distinct clusters. The main differences were assigned to Raman bands that are associated with protein and DNA vibrations at 1004, 1093, and 1664 cm^–1^ [63]. Other studies exploring the non-invasive screening possibility of hematopoietic neoplastic B- and T-cell lines confirmed that Raman spectroscopy is suitable to discriminate between malignant-transformed and healthy cells based on significant spectral differences assigned to decreasing nucleic acid peaks in the diseased cells [64]. Similarly transformed monocytes were distinguishable from their healthy counterparts due to a decrease of DNA signals and a relative increase of protein signals [65].

Advanced studies have focused on more practical approaches for the evaluation of Raman-based cell classification in clinics. Harvey et al. [66, 67] employed experiments on detecting prostate carcinoma cells to classify not only pathological cells, but also cells from different tissue types that may be present in urine. Differences between bladder cancer cells and prostate cancer cells as well as the discrimination of several urethral cell types were emphasized [66, 67]. Other groups focus on employing Raman spectroscopy as a fast and non-invasive screening tool for the detection of multi-drug resistant tumor phenotypes, which might provide relevant information when choosing cancer therapies [68].

### 3.3 Stem cell characterization and monitoring

The non-invasive nature of Raman spectroscopy makes it an ideal tool for the characterization of stem cells and to monitor their differentiation in vitro. First Raman-based stem cell studies aimed to track the unique pluripotent status of embryonic stem cells (ESCs). The Raman spectra of murine and human ESCs were found to be strongly dominated by nucleic acid-specific peaks of DNA (782 cm^–1^) and RNA (813 cm^–1^). A decrease of these specific peaks during differentiation was reported in several studies. It was suggested that the spectra reflected different molecular events connected with stem cell differentiation, including an increase in cell size, increase of tissue-specific proteins, a decrease of the proliferative activity, or a drop of the nuclear RNA content [69, 70]. Furthermore, it was shown that Raman spectral analyses also reflected the heterogeneity of spontaneously differentiated cells [71]. A recent study reported the decrease of RNA signals when murine neuronal stem cells differentiated into glioblastoma cells. Herein, it was suggested that an increase in cell size leads to the dilution of cytoplasmic RNA and thus to a decrease of RNA-specific Raman signals [72].

When comparing Raman spectra of pluripotent murine ESCs and cells of the primitive endoderm, it had been suggested that spectra from primitive endoderm displayed the presence of carbohydrates. This was particularly true for the peak at 937 cm^–1^ [73]. Heterogeneities concerning the glycogen distribution in human ESC colonies were detected in other Raman spectroscopy-based studies and might be correlated to spontaneously differentiating ESCs [74]. Further studies revealed the feasibility of Raman spectroscopy for glycogen quantification [75].

Moreover, Raman spectroscopy was successfully used to discriminate human ESCs from in vitro-differentiated, spontaneously beating cardiac cells. The spectra from ESC-derived cardiac cells revealed spectral markers analogous to those of isolated fetal cardiomyocytes [76]. In successive studies, the Raman spectral analysis was combined with immunofluorescence staining of alpha-actinin to confirm the differentiation status of single cells. Thus, cardiac cells have been successfully discriminated from other ESC-derived cells. The cardiac Raman pattern was unique due to contributions of cellular glycogen as well as specific cardiac proteins [77, 78].

Raman spectroscopic comparison of human ESCs with human MSCs revealed specific peaks assigned to intracellular lipids in ESCs which were related to the ESC-specific pluripotent state [79]. Our studies demonstrated significant differences between the spectra of porcine chondrocytes and porcine MSCs [21], as well as between human primary fibroblasts and human MSCs. [44]. Other studies have focused on the implementation of Raman-based monitoring for the differentiation screening of MSCs in vitro. During osteogenic differentiation, calcium-hydroxyapatite accumulation and crystallinity was detectable due to the unique 950–970 cm^–1^ apatite peak. The carbonate-to-phosphate ratio and the mineral-to-matrix ratio calculated from Raman data were suggested to be a useful quality parameter for mineralization [80, 81]. Moreover, Raman spectroscopy allows the comparison of distinct effects on the osteogenic differentiation such as different cell types [51] or effects due to media supplements [82] as well as continuous real-time monitoring [83].

### 3.4 Real-time screening for toxicity

Raman spectral fingerprinting is highly influenced by the viability of cells. First studies explored changes in Raman spectra of human lymphocytes that were exposed to continuous laser irradiation. A decrease in the overall Raman spectra was detected and found to be especially evident in the 1487 cm^–1^ Raman band, which is assigned to purin and pyrimidin bases [9]. Decreasing DNA and protein peaks were confirmed in studies comparing living and dead cells [84]. Other studies demonstrated that DNA fragmentation induced with Triton X-100 directly correlates with a decrease of the DNA backbone signal at 788 cm^–1^ [85]. Continuous measurements of dying cells demonstrated an increase in protein peaks at the beginning of Triton X-100 incubation. However, as soon as the metabolic activity was significantly reduced, protein-specific signals started to decrease. Progressive cell death was further evident in a shift in width of the amide I peak near 1660 cm^–1^, as well as a rapid decrease in DNA backbone vibrations. Diminishing DNA peaks were related to the fragmentation of DNA [86].

There are many other reports on the distinct effects of cell viability and cell death detected by Raman spectroscopy [87–89]. In various studies, Raman spectroscopy was employed as a screening tool for chemotherapeutics. Raman spectroscopy additionally confirmed that etopisoide, taxol, and doxorubicin treatment leads to chromatin condensation and fragmentation also known in apoptosis [90–94]. Furthermore, the toxicological comparison of different substances showed that Raman spectroscopy discriminated substance-specific mechanisms of cell death [95, 96].

## 4 In vitro analysis of extracellular matrix components – detection of collagens and proteoglycans

Raman spectroscopy has been used to characterize extracellular matrix (ECM) components in tissue samples. Spectra were shown to reflect tissue components such as collagens, elastin, proteoglycans, chondroitin sulfate, and hyaluronic acids [97, 98]. Furthermore, Raman spectroscopy has been shown to be applicable to distinguish between different tissue layers. For example, the discrimination of the skin layers (stratum corneum, epidermis, and dermis) was verified based on their predominant matrix components [99–101]. In these studies, spectra were generated from native, paraffin-embedded, or snap-frozen tissue specimens. In our studies, we have probed native porcine aortic heart valves, which have a highly organized ECM structure with distinct collagen-rich and elastin-rich layers. Disruption and remodeling of the heart valve ECM structure is involved in heart disease states [102]. We detected a decrease in collagen-specific Raman bands due to enzymatic degradation of collagens. We could further show that collagen structure is significantly damaged in cryopreserved heart valves [103]. Others utilized a transgenic mouse model with known modifications to the collagen type II gene that result in the development of a truncated collagen fiber. Employing Raman spectroscopy, the authors found that disordered structures of modified collagen type II are most evident in the amide III peak (1230–1280 cm^–1^) [104]. A special disruption of the collagen structure due to laser ablation and heat was detected in pig cornea using Raman spectroscopy [105].

Raman investigations on the subzones of articular cartilage showed that spectra reflected the distinct distribution and orientation of collagen, proteoglycans, and chondroitin sulfate [52]. The spectra of the deep and middle zones reflected high amounts of collagen; whereas in spectra of the superficial zone, chondroitin sulfate and aggrecan were predominantly detected [52]. Similar findings demonstrated that Raman-based marker-free chemical imaging is a practical method to display the distribution of matrix components in cartilage [106]. Raman spectral changes were also detected in disrupted cartilage. In this study, a significant decrease of the glycosaminoglycan signal contributed to a decrease in the amide III Raman band at 1126 cm^–1^ [107]. Increased spectral signals from proteoglycans and chondroitin sulfate were also reported when probing spinal cord injuries in rats [108].

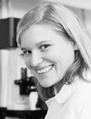

**Eva Brauchle** received her Master of Science in Biomedical Engineering in April 2011. She is currently working on her PhD thesis at the Fraunhofer IGB in Stuttgart, the University Stuttgart and the University Hospital Tübingen, Eberhard Karls University Tübingen, Germany. Her research focus is to use Raman spectroscopy for the non-contact identification and characterization of stem cells and stem cell-derived somatic cells.

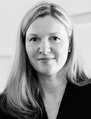

**Katja Schenke-Layland** is the Professor of Biomaterials in the Department of Thoracic and Cardiovascular Surgery and the Inter-University Centre for Medical Technology Stuttgart-Tübingen (IZST) at the University Hospital Tübingen, Eberhard Karls University and the Deputy Head of the Department of Cell and Tissue Engineering at the Fraunhofer IGB Stuttgart, Germany. Her main research focuses on human cardiovascular development, stem cell, and extracellular matrix biology, as well as optical technologies in biomedical applications.

## 5 Conclusions and outlook

In this review we discussed Raman spectroscopy as an in vitro tool for the marker-free biochemical characterization of cells and tissues. Raman spectroscopy was successfully employed to identify and discriminate bacterial strains and is on the verge of being implemented in microbial quality control as well as for the diagnostic of infectious disease. The sensitivity of Raman spectra to metabolic characteristics of microorganisms as well as mammalian cells allows the monitoring of metabolic pathways such as the expression of recombinant proteins. Therefore, Raman spectroscopy may also be implemented as a useful tool for single cell screenings. Microfluidic devices for high-throughput cell identification and cell sorting based on Raman spectra are currently under development [109, 110]. Analyzing metabolic signatures is especially critical for cell-based therapies and tissue engineering approaches. Here, cells need to be identified and analyzed prior transplantation into a patient. So far, there is no appropriate standard method to overcome this analytical challenge. Raman spectroscopy can be performed non-destructively on rare materials such as primary cells and stem cells. Additionally, tracking developmental changes within stem cells using Raman spectroscopy might give new insights into (epi)genetic and metabolic shifts that are interesting for various fields in biomedical research.

Raman-based studies on pathological cells in vitro predominantly focused on the discrimination of tumorigenic cells and non-tumorigenic cells. Most of studies were performed on well-known in vitro cell lines; however, it is assumed that studies on patient cells are much more challenging due to a high heterogeneity of these primary cancer cells [111].

Raman spectroscopy has proven to be sensitive to biochemical alterations that occur during cellular damage and proceeding cell death. However, progression of cell death and the effect of different cytotoxic substances may be cell type-specific and differs in kinetics. Nevertheless, Raman spectroscopy can easily be employed as an assay system to track and compare cytotoxic effects.

Biochemical alterations of extracellular tissue components are involved in many pathological states such as skin aging, arthritis, and heart valve failure. Raman spectroscopy has not yet been explored for all diseases, but may give new insights into dynamic matrix remodeling processes. In the future, Raman spectroscopy might be established as a universal tool for many biomedical applications. The combination of Raman spectroscopy with other optical methods such as coherent anti-Stokes Raman imaging may enhance the content and quality of information [11]. Raman spectroscopy has the potential to reveal insights into known, but also unknown biochemical processes.

## Abbreviations

CHO: Chinese hamster ovary
ESC: embryonic stem cell
HPV: human papilloma virus
INF: interferon
MSC: mesenchymal stem cell
NIR: near-infrared
PC: principal component
PCA: principle component analysis
PHB: polyhydroxyalkanoates
PI: propidium iodide
